# Robotic Partial Cystectomy for Urachal Carcinoma: A Case Report and Review of the Literature

**DOI:** 10.1155/2021/6743515

**Published:** 2021-11-09

**Authors:** Rawad Abou Zahr, Valentin Colinet, Aurore Mattlet, Teddy Jabbour, Romain Diamand

**Affiliations:** ^1^Urology Department, Jules Bordet Institute, Université Libre de Bruxelles, Boulevard de Waterloo 121, 1000 Brussels, Belgium; ^2^Urology Department, Saint George Hospital University Medical Center, University of Balamand, Beirut 1100 2807, Lebanon

## Abstract

Urachal carcinoma is a very rare tumor, commonly found in the urachal remnant connecting the bladder dome to the umbilicus. Diagnosis is often challenging due to the location of the tumor and its late presentation. We hereby report the case of a 49-year-old female where the diagnosis of urachal carcinoma was made and a robotic partial cystectomy associated with en bloc resection of the umbilicus was performed. We aim to present the clinical aspects, presentation, and diagnosis of this rare entity along with a review of the literature.

## 1. Introduction

Embryologically, the urachus connects the allantois to the apex of the bladder; it later degenerates to become the median umbilical ligament. The urachus is located between the transverse fascia anteriorly, the peritoneum posteriorly, and the umbilical arteries laterally. Urachal remnants are found in almost one third of the population [1]. Urachal carcinoma was first reported by Begg in 1930; it is a rare but highly malignant epithelial cancer [2]. Histologically, urachal remnants resemble intestinal epithelium more closely than adjacent urothelium. As such, urachal tumors differ histologically and biologically from other bladder neoplasms, with adenocarcinoma being the most commonly encountered histological subtype [3].

Urachal tumors are rare and only represent less than 1% of bladder tumors and almost 0.01% of adult malignancies [4]. It is more common in men than in women with a ratio of 1.49 : 1, and it mostly presents after the age of 50 [2].

Hematuria especially macroscopic is the major clinical presentation of these tumors; it can be associated with pelvic pain, dysuria, and mucinuria [5]. None of these symptoms is specific to this particular entity of bladder tumor. Hence, the main diagnostic pathway remains the same as for usual bladder tumors which starts by cystoscopy to visualize and locate the tumor. Most lesions are located at the dome and anterior wall of the bladder. Urachal carcinoma can originate at any site between the umbilicus and the dome of the bladder, and thus, it may present with different clinical manifestations [6]. Urine cytology is only positive in 38% of cases which may be because of the extravesical location of the tumor [7]. Computed tomography (CT) and magnetic resonance imaging (MRI) may be used in the clinical evaluation of the tumor to determine the involvement of other organs and can serve to evaluate locoregional extension of the tumor [3, 8].

Biomarkers such as CEA, CA-125, and CA 19-9 may be elevated in 40–60% of patients; however, they are nonspecific and can be elevated in cancers of other origins [9].

## 2. Case Presentation

A 49-year-old female, nonsmoker, presented with persistent hematuria of one-month duration. She reported no pain or dysuria. Microscopic examination of the urine did not show evidence of infection. The patient has a BMI of 24 kg/m^2^; she has a history of 2 normal vaginal deliveries and has no previous abdominal surgeries except for a tubal ligation 10 years ago. The patient has an office job and has no exposure to environmental or workplace toxins. She reported having regular menstrual cycles, was not following up regularly with her gynecologist, and has no family history of cancer.

**Figure 1:**
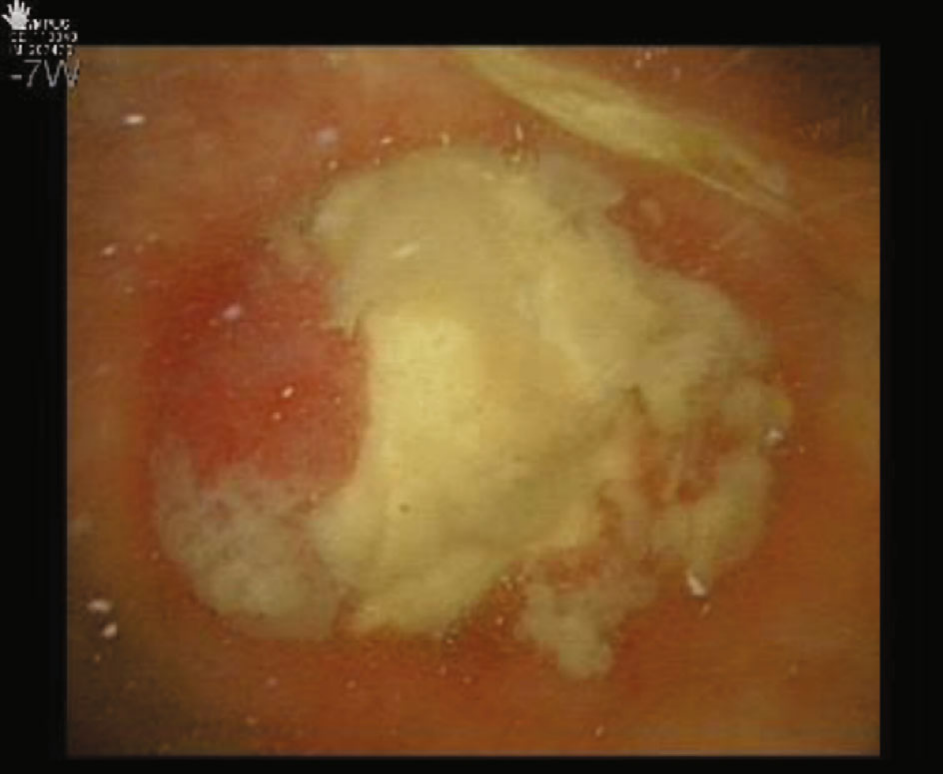
Cystoscopic examination showing a mucinous lesion at the bladder dome.

**Figure 2:**
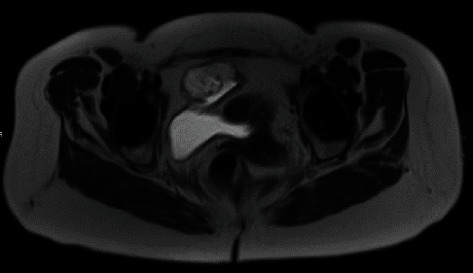
Magnetic resonance imaging showing a lesion at the expense of the bladder wall.

Cystoscopic examination showed a mucinous lesion at the level of the bladder dome (Figure 1), followed by a Triphasic CT scan and MRI confirming the presence of a 6 cm mass at the level of the bladder dome. (Figure 2) A transurethral resection was then performed with the pathology report indicating a mucinous adenocarcinoma.

Due to the rarity of this tumor in the bladder, a complete gastroenterology evaluation to exclude a digestive primary tumor was performed. The gastroscopy and the colonoscopy were both normal.

An abdominal and pelvic MRI (Figure 2) completed the evaluation of this tumor to exclude a possible ovarian or appendicular origin. The appendix and annexes were normal, and the mass was localized in the bladder wall. The intensity of the signal and the contrast enhancement were in favor of a mucinous lesion. It corresponded to stage 2 urachal carcinoma according to the Mayo staging system. Tumor markers CA125 and CA19/9 were negative.

After a multidisciplinary team meeting, a robotic partial cystectomy with en bloc resection of the urachus and umbilicus was performed. Pelvic lymphadenectomy was excluded. The final evaluation of this tumor was made by laparoscopic exploration to exclude any potential peritoneal carcinomatosis.

**Figure 3:**
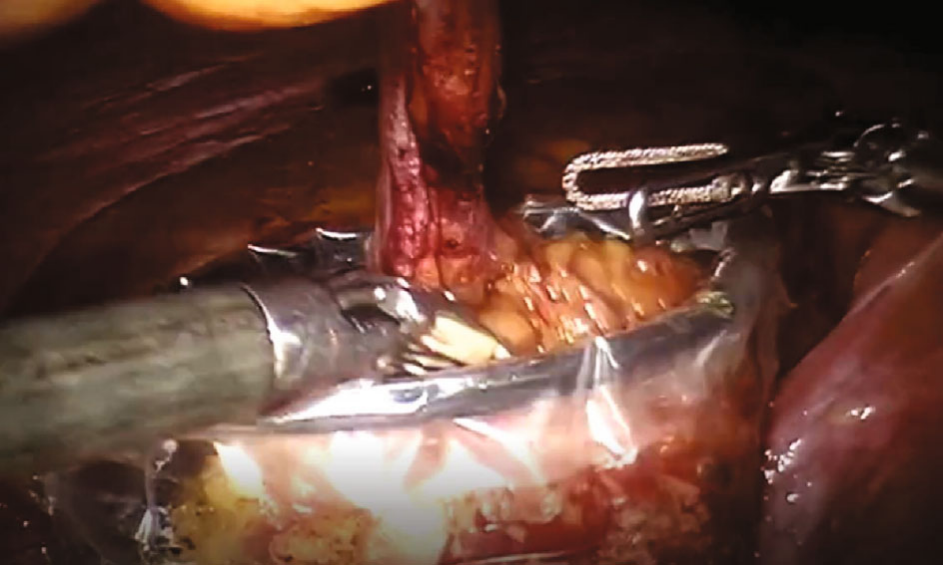
En bloc resection of bladder tumor with umbilicus.

The approach chosen for the intervention was minimally invasive, and hence, a robotic-assisted partial cystectomy with en bloc resection of the umbilicus was successfully performed (Figure 3). En bloc resection with the umbilicus, in addition to having a foley catheter in place during the procedure, limits tumor spread and spillage.

**Figure 4:**
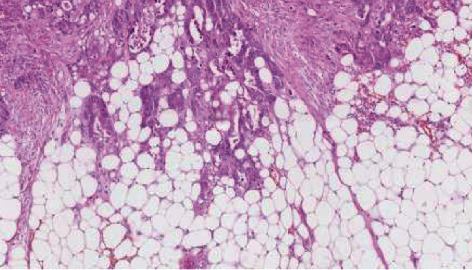
Focal conventional adenocarcinoma infiltrating perivesical fat.

**Figure 5:**
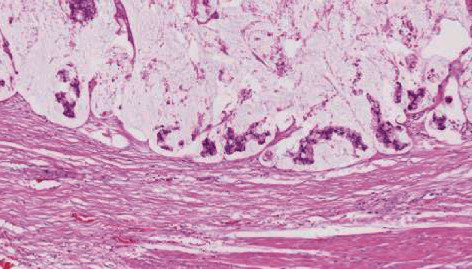
Predominant mucinous adenocarcinoma, with strands of glandular cells floating in abundant extracellular mucin.

The operative piece measured 9 × 5 × 4.5 cm, and the final histology confirmed a mucinous adenocarcinoma of the urachus (Figures 4 and 5). Margins were negative, and there was no sign of lymphovascular invasion; the pathological TNM staging was pT3bNx.

Immunohistochemistry tests were made on the operative piece; no microsatellite instability (MSI) was found. A pathologic mutation was detected in the TP53 gene.

Patient had an uneventful postoperative stay; she was discharged at day 5 postop, and a foley catheter left in place was removed at 10 days. No adjuvant therapy was given to our patient. Follow-up of her disease comprises of CT scan every 3 months for the first year. Her follow-up is negative for any recurrences to this date.

## 3. Discussion

Urachal carcinoma is an uncommon tumor arising mostly from the urachal remnant. Due to its localization in the Retzius space, symptoms of urachal carcinoma do not appear in early stages of the disease [1]. Due to this, patients usually present with advanced or metastatic disease at presentation. The patient depicted in the case above presented with a sole complaint of gross hematuria of one-month duration and was found to have local disease with no extravesical extension.

There are two main staging systems to describe urachal carcinoma, the Mayo staging system and the Sheldon staging system. The former newer system was shown to be less complicated; however, both systems predicted cancer-specific mortality comparably [7]. Dhillon et al. showed that urachal adenocarcinoma is associated with a better prognosis than bladder urothelial carcinoma, with a cancer-specific survival of 45 months versus 27 months when compared at the same stage [1]. Tumor size and the presence of signet ring differentiation were controversially reported as prognostic factors, whereas mucinous tumor phenotype had no prognostic effect on survival [5].

Given that most urachal cancers are resectable, surgery remains the main treatment modality. Surgical treatment encompasses either partial cystectomy, radical cystoprostatectomy, or cystectomy [1]. It is worth to note that according to Gopalan et al., the incidence of local recurrence was higher in patients undergoing partial rather than radical treatment [10]. A defined surgical protocol is yet to be consolidated; however, surgical management should depend on tumor resectability, ability to obtain clear margins and patient factors. In a 2016 meta-analysis comprising more than 1000 patients, Szarvas et al. demonstrated that in cases where data on umbilectomy was available, 67% of these patients underwent umbilectomy in accordance with recommendations of most authors [5]. In fact, in their large retrospective review, Ashley et al. demonstrated that failure to perform umbilectomy was associated with increased cancer-specific mortality based on univariate analysis [7]. However, another review by Siefker-Radtke et al. concluded that umbilectomy in itself was not found to be a statistically significant factor in survival [3]. Different surgical approaches vary due to the lack of clear guideline recommendations. Hence, data regarding the benefit of umbilectomy has to be clearly highlighted to encourage clinicians to adopt this as an integral part of the surgical procedure.

The prognostic effect of lymph node dissection (LND) remains controversial at the time of surgery. However, its utility might be explained given the fact that patients with positive lymph nodes at the time of surgery carry a poorer survival outcome similar to those with distant metastasis [5]. This demonstrates a possible role for LND in accurate staging which might change treatment decisions depending on positivity. In the 2016 meta-analysis by Szarvas et al., only 17% of patients that underwent LND were found to be positive [11]. Such patients were allocated to LND given high-risk features and still yielded relatively low rates of positivity. Therefore, the benefit of LND in patients with urachal carcinoma still shows low levels of prognostic utility [5]. Future studies might be needed to demonstrate the utility of lymphadenectomy in patients with urachal cancer.

Given the fact that urachal cancer is relatively a clinically silent disease, >20% of patients present with metastatic disease at presentation. With urachal cancer histology being similar to that of colon cancer, 5FU combination chemotherapy regimens demonstrated most benefit in patients with recurrent or metastatic disease [5].

To demonstrate the oncological safety of the surgical technique, Knoedler et al. showed that when compared to patients who underwent radical cystectomy for all bladder cancers, patients that underwent partial cystectomy had no difference in metastasis-free or cancer specific survival. However, patients who opt to partial cystectomy remain at risk of local recurrence [12]. Given that the risk of “seeding” in adenocarcinoma remains significantly less than that of urothelial carcinoma of the bladder. In their retrospective study, Golombos et al. showed that adhering to traditional indications of ruling out CIS before partial cystectomy and having a tumor in a favorable location, this technique demonstrates both favorable recurrence and survival rates [11]. In our case, care was taken to remove the specimen en bloc with the umbilicus after suspending the whole specimen (Figure 3); resection was also done on an empty bladder with a foley catheter in place as to avoid urine spillage as much as possible.

## 4. Conclusion

Urachal carcinoma is a rare nonurothelial malignancy arising at the dome or anterior wall of the bladder. Given that the most common histology is adenocarcinoma, this highlights the importance of accurate diagnosis since a different staging and treatment path will be taken compared to urothelial cancer. Surgery remains the main modality of treatment either radical or partial with most surgeons advocating an umbilectomy en bloc. Lymph node dissection remains a controversial topic given the low prognostic yield available in the current literature. Robot-assisted partial cystectomy with en bloc umbilectomy seems to be an excellent treatment option for patients with evidence of localized disease with excellent oncological and cosmetic outcomes.

## Data Availability

All data used in this case report are included in the manuscript.
